# Next generation sequencing: a possible answer to sudden unexplained deaths in a young South African cohort?

**DOI:** 10.1007/s12024-025-00944-6

**Published:** 2025-02-03

**Authors:** Barbara Stroh van Deventer, Lorraine du Toit-Prinsloo, Chantal van Niekerk

**Affiliations:** 1 Department of Forensic Medicine, University of Pretoria, R4-41 Pathology Building Prinshof Campus, Pretoria, 0002 South Africa; 2 Department of Chemical Pathology, Faculty of Health Sciences, University of Pretoria / NHLS, Pretoria, South Africa; 3 New South Wales Health Pathology, Forensic and Analytical Science Service (FASS), New Castle, New South Wales Australia; 4 Department of Chemical Pathology, National Health Laboratory Services, Pretoria, Australia

**Keywords:** Inherited cardiac arrhythmogenic disorders, Molecular autopsy, Next-generation sequencing (NGS), Sudden unexpected death (SUD), Sudden unexplained infant death (SUID)

## Abstract

Sudden cardiac death (SCD) is a major public health concern. In sub-Saharan Africa (SSA), including South Africa, there is a lack of reliable statistics on the incidence of SCD, even though there has been a fourfold increase in noncommunicable diseases (NCD), particularly cardiovascular diseases (CVD). Sudden cardiac death contributes to an estimated 50% of all cardiovascular deaths, which highlights South Africa’s need for research into better detection, treatment and prevention. This study aimed to identify an inherited cardiac arrhythmogenic disorder, linked to variants in cardiomyopathy- and arrhythmia-related genes, as a potential contributing factor to sudden cardiac deaths. DNA was extracted from blood samples collected at autopsy of 51 sudden unexpected death (SUD) cases, and subjected to next-generation sequencing (NGS) of 49 genes linked to inherited cardiac arrhythmogenic disorders. Variants were annotated and interpretated for clinical significance using the Galaxy bioinformatic platform. In total, 175 different missense variants were identified in the study population (*n* = 51). Of these, 92.5% (162/175) were known, documented variants, and the remaining 7.4% (13/175) were considered novel. Of the known variants, 78.4% (127/162) were of benign/likely benign significance, 20.4% (33/162) were variants of unknown significance (VUS), and 1.2% (2/162) was pathogenic. The 13 novel variants were analysed using online prediction software, with 92.3% (12/13) predicted to be likely benign and 7.7% (1/13) grouped into the VUS category. Post-mortem genetic testing provided evidence of a genetic arrhythmic/cardiac conduction disorder as the probable pathogenic basis for approximately 4% (2/51) of sudden unexpected death (SUD) cases.

**Supplementary Information:**

The online version contains supplementary material available at 10.1007/s12024-025-00944-6.

## Introduction

Post-mortem genetic testing, also termed the molecular autopsy, is invaluable in a forensic setting and is used to identify genetic variants associated with, or causative of, a disease. This, in turn, may lead to a better understanding of the cause of death, specifically in cases of sudden unexpected death (SUD) [1, 2]. A SUD is defined as “a natural, unexpected fatal event that occurs within one hour of the beginning of symptoms, or 24 hours in cases where the death was unwitnessed, in an apparently healthy subject or in one whose disease was not so severe that such an abrupt outcome could have been predicted” [3]. Genetic testing is costly and thus often unavailable in economic - and resource-constrained countries.

Cardiac related SUDs [termed sudden cardiac deaths (SCDs)] are deemed a major public health concern and better detection, treatment, and prevention strategies are being developed [4–6]. The estimated global incidence of SCD ranges between 15 and 159 cases per 100, 000 people per year, with approximately four to five million global deaths per annum [6,77. These constitute 20% of all deaths in Western societies [6, 7].

The cause of SCDs is greatly dependent on age, with ischaemic heart disease (IHD) the most common cause of death in older populations [8]. In younger populations (≤ 45 years), up to 90% of cases are caused by inherited cardiac diseases, mostly cardiomyopathies and arrhythmogenic disorders [2, 9]. Microstructural cardiomyopathy changes can be overlooked at autopsy, whereas arrhythmogenic disorders can neither be macro- or microscopically diagnosed [10, 11]. Research has shown that, in the absence of post mortem genetic testing, up to 50% of these SCDs remain unexplained after an autopsy investigation [12]. Consequently, many first world countries have implemented post mortem genetic testing as a routine procedure when investigating SCDs in their young population [5, 6].

There is a lack of reliable statistics in sub-Saharan Africa (SSA) (including South Africa), on the incidence of SCDs. This is despite reports indicating a fourfold increase in noncommunicable diseases (NCD), primarily driven by cardiovascular diseases (CVD) [4, 13]. CVDs are the second biggest killer in SSA, with the mean age of onset recorded as the youngest in the world [13, 14] Approximately 2,000 young South Africans die suddenly each year, with the cause of death remaining unexplained [14, 15].

Under South African law, all SUD cases must undergo a comprehensive medico-legal death investigation to determine the cause and manner of death [16]. However, post mortem genetic testing has not yet been implemented in South African mortuaries. Cost is a factor, but this lack of implementation is also due to limited research in this field. International studies highlight the underrepresentation of African cohorts in health research, with a particular lack of genetic data such as genetic causes of SCDs [17]. Aside from one study involving a small cohort of sudden unexplained infant deaths (SUID), no other research in South Africa has investigated the potential role of inherited cardiac arrhythmogenic disorders in unexplained SCDs among the young [15]. Therefore, this study aimed to assess the prevalence of genetic variants in 49 major genes associated with inherited cardiac arrhythmogenic disorders in unexplained SUD cases among the young, admitted for medico-legal autopsy at a prominent South African medico-legal laboratory (MLL).

## Methods

### Ethical approval

Ethics approval for this study was obtained from the Faculty of Health Sciences Research Ethics Committee, University of Pretoria (495/2017). The South African Inquests Act 58 of 1959 allows for any ancillary investigations that can assist in determining the cause of death.

### Study cohort

A prospective genetic study was conducted on 51 SUD cases that were admitted to the MLL. In all included SUD cases (age range between one and 45 years) the cause of death could not be determined after a full medico-legal death investigation had been performed. This covered a full autopsy, death scene investigation, review of available medical history as well as all appropriate ancillary investigations (e.g., virology, toxicology, histology, microbiology etc.) Peripheral blood samples were collected at autopsy into two 5 mL EDTA tubes and immediately stored at -80 °C until DNA extraction could be performed.

### Genetic testing

DNA was extracted from post mortem blood samples using the QIAamp DNA Blood Mini Kit (Qiagen, Hilden, Germany) according to prescribed guidelines provided by the manufacturer. DNA samples were fluorometrically quantified and diluted to 10 ng per primer pool, using the Qubit dsDNA HS Assay kit on the Qubit 3.0 Fluorometer (ThermoFisher, Waltham, Massachusetts). For next-generation sequencing (NGS) (see Supplemental S1), the AmpliSeq On-Demand DNA Panel (DesignStudio™ software, Illumina, San Diego, California), was designed for the targeting of 49 genes linked to inherited cardiac arrhythmogenic disorders (see Supplemental Table 1). Libraries were prepared using the AmpliSeq Library Plus kit, and subsequent quality was analysed using the Agilent 2100 Bioanalyzer and the Agilent DNA 1000 kit (Agilent Technologies, Santa Clara, California). Quantification was performed (Qubit 3.0 Fluorometer and Qubit DNA HS Assay kit). Pooled libraries were diluted to a final loading concentration of 1.5 pM, followed by sequencing using the v2.5 (300 cycles, 2 × 150 bp paired end reads) high output kit on the Illumina NextSeq 550 platform. A genomic DNA reference sample, NA12878 (Coriell Institute, Camden, NJ), was included in every library preparation and served as a control in each sequencing run.

### Bioinformatic analysis

Sequencing analysis was performed on the open-source Galaxy bioinformatics platform (usegalaxy.com). FASTQ sequencing files were downloaded, quality was assessed using FastQC (v.1.11; http://www.bioinformatics.babraham.ac.uk/projects/fastqc/) and the Trimmomatic tool (v.0.36; available at http://www.usadellab.org/cms/index.php?page=trimmomatic) was used for trimming and filtering. Sequences with an average quality below 20 (within a four bp window) were cut. Only reads with an average quality threshold of 30 (Q > = 30) and a minimum length of 80 bp were kept for further analysis. Post-trimming reads were aligned to the reference human genome (GRCh37, hg19) using the Burrows-Wheeler Alignment – Maximal Exact Match (BWA-MEM) tool (v.0.7.18; http://bio-bwa.sourceforge.net). Individual reads were labelled using the read group tag in the “sequence alignment map / binary alignment map (SAM / BAM) specification.” Picard was used for a clean-up of BAM files and the removal of duplicate reads (v.3.2.0; https://broadinstitute.github.io/picard/). Reads were further filtered (SAMtools v.1.8; http://www.htslib.org/) keeping only those with a mapping quality (MAPQ) > = 30 and mapped in a proper pair. Finally, all files were merged into one BAM file and visualized [JBrowse genome browser v.1.16.11 (https://gmod.or/wiki/JBrowse) & University of California Santa Cruz (UCSC) genome browser (https://genome.ucsc.edu/)]. Variant calling was performed (FreeBayes v.1.3.6; https://github.com/ekg.freebayes) to generate a variant call format (VCF) file. Variant representation was simplified (VCFAllelicPrimitives tool v.1.0.0_rc3; https://github.com/vcflib/vcflib), followed by variant annotation (SnpEff prediction tool v.4.3.1t; snpeff.sourceforge.net). Variants were filtered (SnpSiftFilter tool v4.3.1t; snpeff.sourceforge.net/SnpSift.html), and all reads that met the following criteria were kept: mapping quality (MQ) > = 60, read depth (DP) > = 20, quality by depth (QD) > 2, genotype quality (GQ) > = 20 and for heterozygous variants an allele balance (AB) between 0.25 and 0.75. Finally, only missense, nonsense, insertion / deletion (INDEL), frameshift and / or splice site variants were retained and visualized (JBrowse genome browser).

The resulting VCF files (filtered variants) were uploaded to two different databases (Jpopgen – dbNSFP and Ensemble variant effect predictor) for further functional prediction and annotation. The Human Genome Variant Society (HGVS) guidelines were followed for variant nomenclature. Several different population databases were compared in relation to the allele frequency (AF) of identified variants (Table 1). A combination of eight different in-silico tools, based on different methodologies, were used for functional effect prediction (Table 1). UniProt was used for the location of the protein domain / region, and Blocks Substitution Matrix (BLOSUM) for the amino acid substitution conservation score. Sequence conservation was measured using the Genomic Evolutionary Rate Profiling (GERP) score as well as the PhyloP100way score. APPRIS was used to annotate alternative spliced transcripts, with splice site predictions scores generated by SpliceAI.

### Variant classification

Variants were interpreted and classified, according to the American College of Medical Genetics (ACMG) and the Association for Molecular Pathology (AMP) guidelines, into one of five groups: (1) pathogenic, (2) likely pathogenic (LP), (3) variant of unknown significance (VUS), (4) likely benign (LB) or (5) benign [18]. The number of criteria across different levels of evidence of pathogenicity was investigated for each novel, VUS, LP and / or pathogenic variant. In addition to in-silico prediction tools, several variant databases, as well as published literature, were searched manually with particular emphasis on functional studies, position in a critical / functional domain of the protein, as well as the mechanism of disease associated with the particular gene.

## Results

### Demographics and history

Demographic details of all cases can be seen in Supplemental Table 2. The study cohort consisted of 51 unexplained sudden deaths with an average age of 28 years. The cases were divided into two age categories in order to be consistent with the literature (1–18 years; 19–45 years). Most of the cases were between 19 and 45 years of age (43/51; 84%) with only 8/51 cases (16%) falling into the younger age group. Most cases were male (35/51; 68%). In only three cases a personal medical history of seizures and palpitations was documented, and no case had documentation showing a family history of syncope or sudden death.

### Genetic results

**Fig. 1:**
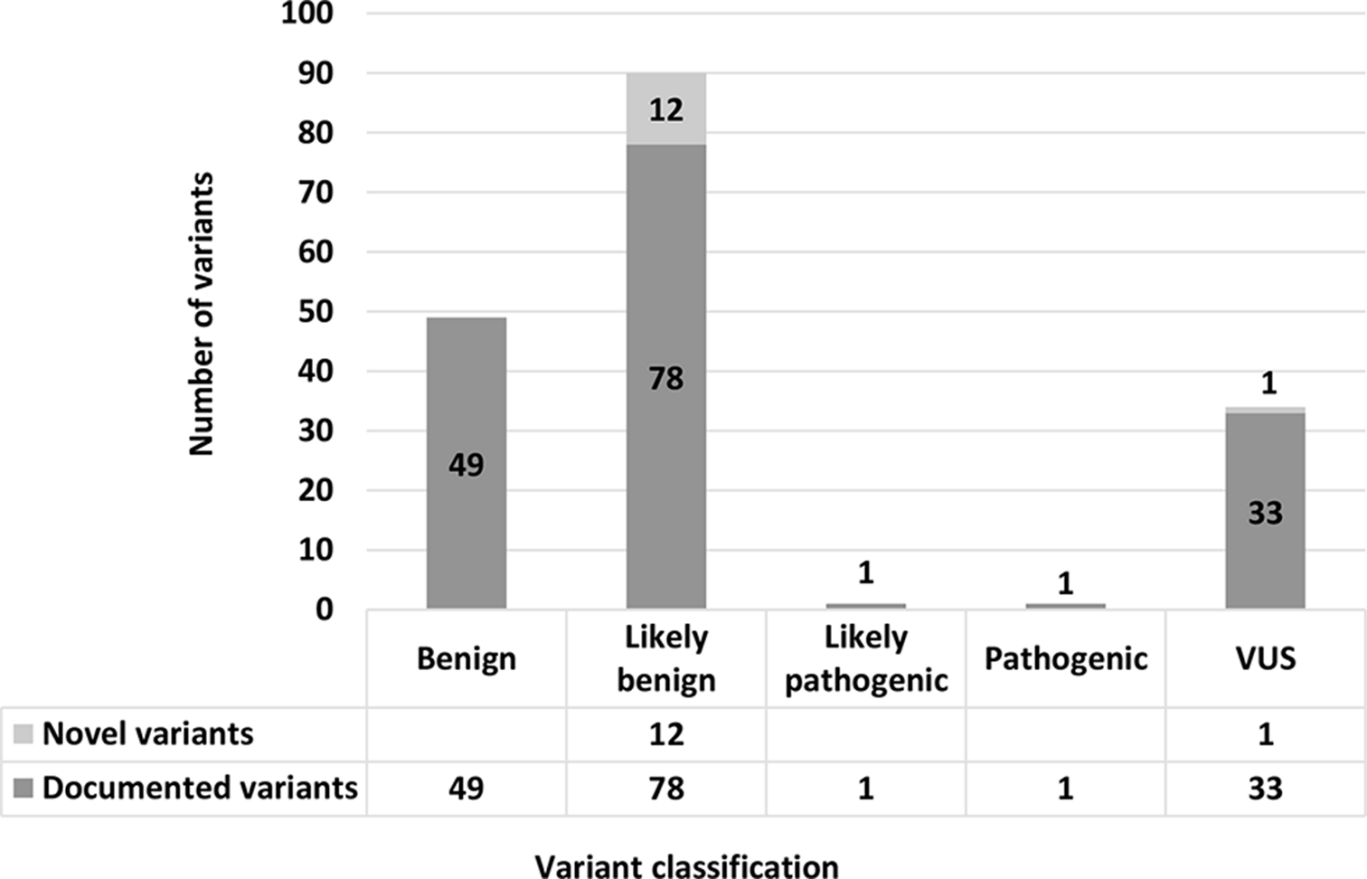
Classification of all identified missense variants. A total of 175 different missense variants were identified among the entire study population (*n* = 51); 162 were known, documented variants whereas the remaining 13 were novel. Of the known variants, 49 were documented to be of benign significance, 78 of LB significance, 33 of unknown significance (VUS), one LP and one of pathogenic significance. Of the novel variants, 12 were predicted to be likely benign whereas the remaining one was considered a VUS

NGS identified a total of 175 different missense variants (Fig. 1; Supplemental Table 3) in the study population (*n* = 51). Of these, 92.5% (162/175) were known, documented variants, and the remaining 7.4% (13/175) were considered novel. Of the known variants, 78.4% (127/162) were of benign/LB significance, 20.3% (33/162) were VUSs, and 1.2% (2/162) was pathogenic. The 13 novel variants were analysed using online prediction software, with 92.3% (12/13) predicted to be LB and 7.7% (1/13) grouped into the VUS category. All 51 cases were found to carry multiple missense variants, most of them LB. In 72.5% (37/51) one or more VUS’s were identified. In approximately 4% (2/51) a variant classified as either pathogenic or LP was found.

#### Variants with moderate and supporting evidence of pathogenicity

For five variants, all eight in-silico tools predicted a deleterious effect on the gene / gene product, each in a different case and each in a different gene (Table 2). This warranted further evaluation of each variant. Four of these variants were documented, one was novel. Three variants (3/5) (two known, one novel) identified in three different cases (3/51) had moderate and supporting evidence of pathogenicity, which may point to disease development. However, due to a lack of supporting data (population frequencies, functional studies, family segregation etc.) these variants all remain classified as a VUS. The two remaining variants (2/5) were classified as pathogenic and LP respectively.

**Table 1:** List of in-silico algorithms and population databases used in variant annotation and functional effect prediction

In-silico algorithm	Population database
FATHMM (http://fathmm.biocompute.org.uk)	Exome Aggregation Consortium (ExAC) http://exac.broadinstitute.org/
MutationAssessor (http://mutationassessor.org)	Exome Variant Server (EVS) http://evs.gs.washington.edu/EVS
MutationTaster (http://www.mutationtaster.org)	1000 Genomes http://browser.1000genomes.org
PolyPhen-2 HDIV (http://genetics.bwh.harvard.edu/pph2)	Genome Aggregation Database (gnomAD) http://gnomad.broadinstitute.org
PolyPhen-2 HVAR (http://genetics.bwh.harvard.edu/pph2)	dbSNP http://www.ncbi.nlm.nih.gov.snp
PROVEAN (http://provean.jcvi.org/index.php)	dbVar http://www.ncbi.nlm.nih.gov/dbvar
LRT (http://genome.cshlp.org/content/19/9/1553	
SIFT (http://sift.jvci.org)	

**Table 2:** Summary of six variants subjected to further evaluation of pathogenicity

Case number		Age	Sex	Activity at TOD	Variant	Gene	Exon	Reference number	Variant classification	Associated disease	
Case 6		35 y	M	Exertion	c.8004G > C (p.Cys2668Trp)	*RYR2*	53	RCV004521091.1	VUS	CPVT	
Case 10		24 y	M	Unknown	c.500T > C (p.Lys167Arg)	*SNTA1*	3	rs932909554	VUS	LQTS	
Case 18		38 y	M	Exertion	c.954 C > A (p.Trp318Cys)	*CASQ2*	10	Novel	VUS	CPVT	
Case 32		23 y	M	Rest	c.226 C > T (p.Asp76Asn)	*KCNE1*	3	rs74315445	Pathogenic	LQTS, BrS, ARVC	
Case 51		28 y	M	Rest	c.40G > A (p.Arg14Cys)	*SCN10A*	1	rs750771811	Likely pathogenic	BrS	

Abbreviations: y = years; M = male; CPVT = catecholaminergic polymorphic ventricular tachycardia; LQTS = long QT syndrome; ARVC = arrhythmogenic right ventricular dysplasia; BrS = Brugada syndrome

## Discussion

International research has shown that in up to 40% of SUD cases, an inherited cardiac arrhythmogenic disorder could have been the cause of death [1, 2]. Genetic variants may, however, differ between populations of various countries or regions.

In this study, Case 6 was that of a 35-year-old South African male who suddenly collapsed while walking down the street. Emergency medical services (EMS) declared him dead on the scene. The autopsy revealed no other injuries or signs of pre-existing disease apart from three small superficial abrasions on the left anterolateral aspect of the forehead and temporal region of the face. All ancillary investigations were normal, and with a lack of any clinical history, the ultimate cause of death remained undetermined. Following NGS, a known heterozygous missense variant, (p.Cys2668Trp; ClinVar accession RCV004521091.1), was identified in exon 53 of the *RyR2* gene. This variant is classified on ClinVar as a VUS related to a cardiovascular phenotype (PP3). Conservation scores indicate that it causes a non-conservative amino acid substitution (Cysteine to Tryptophan) within a highly conserved region of the protein (PM1 + PP3). Additionally, eight different in-silico tools predicted a deleterious impact on the gene or its product (PP3).

This variant is located just outside one of the four established *RyR2* hotspots (exons 44–50) but remains within the bridging solenoid (BSol), a major structural domain of the protein [15, 16]. Reports have linked variants in the BSol and N-terminal domains, which form the RyR2 interprotomer contact domain, in affecting channel pore opening [17, 19]. Exercise or stress can trigger an increase in sarcoplasmic reticulum Ca2 + release and so raise the risk of delayed onset depolarization (DOD) and fatal ventricular arrhythmias [17, 20]. In Case 6, records indicate light exercise prior to death, supporting the relevance of a variant in a gene known for exercise-induced ventricular tachycardias.

### Case 10

involved a 24-year-old South African male who, like Case 6, suddenly collapsed while walking in a shopping mall. He was pronounced dead at the scene before any emergency treatment could be administered. The autopsy revealed no external injuries, and internal examination showed only non-specific lung and visceral organ congestion. Ancillary investigations returned normal results, leaving the cause of death undetermined. Post-mortem genetic testing identified a known VUS in exon 3 of the *SNTA1* gene (heterozygous c.500T > C; p.Lys167Arg; rs932909554). All eight in-silico tools predicted a deleterious functional effect, supported by conservation scores indicating a non-conservative amino acid change within a conserved protein domain. Additionally, a global mean allele frequency (MAF) of 0.00007 and an African MAF of 0.0003 were reported, providing moderate and supporting evidence of pathogenicity (PP3 + PM1).

The *SNTA1* gene encodes the heart-expressed syntrophin isoform, a channel-interacting protein that regulates ion channel gating kinetics, particularly the SCN5A channel, through connections to various intracellular pathways [21, 22]. The p.Lys167Arg variant found in Case 10 was located in the PDZ domain of the *SNTA1* gene, which is crucial for regulating the cardiac sodium (SCN5A) channel by binding to its C-terminus [21, 22]. SNTA1 has also been shown to play a key role in the genetic regulation of ventricular cardiac muscle cell membrane repolarization, with variants linked to atrial fibrillation and long QT syndrome (LQTS) [21–23].

### Case 18

was a 38-year-old South African male who collapsed during a casual soccer match in a park. He was declared dead on the scene after numerous attempts by EMS failed to revive him. At autopsy, no external injuries to the body were noted. An internal examination only revealed non-specific congestion of the brain, with no signs of injury or underlying disease in the other organs. Toxicology showed the presence of cannabis; but this was not considered to be the cause of death. All additional ancillary tests were normal, with no personal and / or family medical history of the deceased available. The cause of death remained unexplained.

A novel heterozygous c.954 C > G (p.Trp318Cys) VUS was found in exon 10 of the *CASQ2* gene after NGS analyses. This non-conservative substitution of tryptophan with cysteine occurred in a highly conserved protein region and is associated with a cardiovascular phenotype. This VUS has no reported MAF, and all eight in-silico prediction tools indicated a deleterious effect on the gene and its product, with moderate supporting evidence of pathogenicity (PM1 + PP3). The affected gene (*CASQ2*) encodes a crucial calcium-binding protein located in the sarcoplasmic reticulum responsible for calcium buffering and regulation of calcium release channels in cardiac muscle cells [24, 25]. The protein comprises three thioredoxin-like domains enclosing a negatively charged hydrophilic core, as well as N-terminal and C-terminal (tail) regions [26]. The p.Trp318Cys variant is situated within the conserved helical portion of Domain III, near the C-tail. This domain is essential for SR targeting and *CASQ2* polymerization, with variants in this region linked to catecholaminergic polymorphic ventricular tachycardia (CPVT) [25, 26]. Lahat et al. [25] reported the p.Asp307His missense variant in an Israeli family with CPVT, also located in Domain III of the *CASQ2* gene.

### Case 51

was a 28-year-old South African male who, as a passenger in his friend’s car, suddenly started complaining of not feeling well and rapidly lost consciousness. He was rushed to the hospital, where he was declared dead upon arrival. Personal medical history indicated a visit to a general medical practitioner one week prior to his death, with complaints of flu-like symptoms. He received general medication for the relief of these symptoms and family members said he had no further complaints for the remainder of that week. No history or signs of drug use were reported. At autopsy, an external examination of the body revealed no injuries, with only signs of medical intervention recorded. Macroscopic and microscopic autopsy findings only included non-specific generalised organ congestion, with no injuries or comorbid illnesses noted. A full medico-legal death investigation failed to determine a cause of death.

A known heterozygous p.Arg14Cys (rs750771811) variant was found in the *SCN10A* gene after genetic analysis. This variant was considered LP as it was rare with a MAF of 0.000020 and located in a critical functional domain of the gene (PS4 + PM1), was a non-conservative amino acid change, with a large physicochemical difference in the same position as another documented pathogenic missense variant (PM5), and eight different in-silico tools all predicting a deleterious effect on the gene/gene product (PP3). This variant was documented in the Forensics Science International: Genetics journal as a novel LP variant (PP5) [1].

The *SCN10A* gene encodes for the tetrodotoxin-resistant voltage-gated sodium channel α-subunit 10, which is expressed in the atrial end ventricular myocytes and is located next to the *SCN5A* gene on chromosome 3, sharing 70.4% similar amino acid sequences [27, 28]. The SCN10A channel plays an important role in the electrical function of the heart. Dependent on the voltage difference across the cardiomyocyte’s membrane, the SCN10A channel mediates its sodium ion permeability, and is responsible for the initiation and propagation of the cardiac action potential [29–31]. The SCN10A channel consists of a cytoplasmic N-terminus, four homologous domains (each consisting of six α-helical transmembrane segments) and a cytoplasmic C-terminus [29, 30]. The variant found in this case was identified in exon 1 and located in the cytoplasmic N-terminus of the protein. Variants located in the N-terminus and C-terminus of the channel have been associated with Brugada syndrome (BrS), characterised by a reduction in the late sodium current and a slowing in the action potential firing, resulting in cardiac arrhythmias and SCD [27, 31].

Three different variants, all linked to BrS and / or SCD, have been reported at the Arg amino acid position 14 (p.Arg14) of the *SCN10A* gene [1, 27, 29]. Zhang et al. [27]. identified the p.Arg14His variant in a case of SUD in the Chinese population, which they considered to be LP and the genetic cause of death. Hu et al. [29]. identified the p.Arg14Leu variant in an American family where a diagnosis of BrS was made during a bout of fever in the proband. Functional expression studies showed that this variant causes a significant reduction in sodium channel availability, leading to a positive shift of half-activation voltage, ultimately reducing the cardiomyocyte’s excitability, and initiating cardiac arrhythmias [29]. The third variant, a homozygous p.Arg14Cys, was reported by Heathfield et al. [1]. in a two-month old male SUID case in South Africa. This homozygous variant was the same as the heterozygous variant found in Case 51. No cause of death could be established, with only a history of flu-like symptoms reported. The variant was classified as LP and the probable cause of SUD.

### Case 32

was that of a 23-year-old South African male who was found unresponsive in his home. Shortly after EMS arrival, he was declared dead on the scene. At autopsy, no injuries were noted upon external examination of the body. An internal investigation revealed non-specific generalised organ congestion. As a result of the non-specific autopsy findings and negative results from all ancillary investigations, no definitive cause of death could be ascertained. Subsequently, a known p.Asp76Asn variant (heterozygous) in the *KCNE1* gene was identified and considered pathogenic as it was highly conserved across species and found in a well-established functional domain (PM1), it was rare with an African MAF of 0.00000 and global MAF of 0.00009 (PS4, PM2), there was a large physiochemical difference between amino acids, with eight different in silico tools predicting pathogenicity (PP3), and three international clinical laboratories classified it as pathogenic in databases, with functional studies and family segregation supporting the evidence (PS3, PP1 and PP5) [32].

The *KCNE1* gene encodes for a small transmembrane modulatory subunit, which binds to the KCNQ1 protein to form a voltage-gated ion channel complex and is expressed in the cardiac ventricular myocytes [33, 34]. This complex forms a slowly activating and slowly deactivating cardiac delayed rectifier current (I_Ks_) that is critical in regulating the cardiac action potential [33, 35]. The KCNE1 protein consists of an extracellular N-terminus, a single helical transmembrane domain, and a highly conserved intracellular C-terminus. It is the C-terminus of the KCNE1, specifically the region between amino acid 70 and 81 that binds to the C-terminus of the KCNQ1 protein to form the potassium channel complex [36, 37]. The p.Asp76Asn variant identified in this study was located in this critical region of the KCNE1 C-terminus. When bound to the KCNQ1 protein, KCNE1 modulates the physical properties of the channel, resulting in an increase in the outward current amplitude and thereby slowing its ultimate activation [33, 36, 37]. Variants located in the C-terminus of the *KCNE1* gene, especially the p.Asp76Asn, have been linked to LQTS-diagnosed patients and SCDs [36]. Studies have shown that variants in the critical region of the KCNE1 C-terminus, shift the KCNQ1’s voltage dependence of activation to more depolarising (positive) voltages, causing a reduction in potassium channel conductance (33–35). The p.Asp76Asn variant has specifically been shown to reduce the outward I_Ks_ current density, causing a delay in repolarisation and ultimately prolonging the cardiac action potential, leading to an increase in susceptibility to cardiac arrhythmias and SCD [33, 35, 37].

Many developed countries have included molecular testing as standard when investigating unexplained sudden deaths with a suspected cardiac arrhythmogenic disorder [38]. To our knowledge, this is the first molecular study analysing 49 genes related to inherited cardiac arrhythmias in a South African cohort of SUDs in individuals aged one to 45 years. SUIDs were excluded from this study, since this category is typically addressed independently due to its complexity, which includes a wide range of diseases, the inherent vulnerability of infants, and various external stressors. Overall, pathogenic or likely pathogenic variants were identified in only two cases (approximately 4%), a rate lower compared to other studies [2, 8, 11–12]. This may be attributed to differences in cohort composition, the size of gene panels used for NGS, or the criteria employed for variant filtering and classification. The unique genetic diversity of South Africa is underscored in this study by the identification of 13/175 novel missense variants and one LP variant, previously reported in a single other South African case [1, 13, 39]. This highlights the importance of conducting more molecular research focused on African-specific populations to enhance global public health outcomes. Genetic analysis of SUD cases has significant clinical implications for diagnosing and treating at-risk family members, with the latter being linked to significantly reduced mortality [3, 40].

## Conclusion

In this study, 175 missense variants were identified via NGS in a cohort of 51 young South African SUD cases. Of these, 92.5% (162/175) were known, documented variants, and the remaining 7.4% (13/175) were considered novel. VUSs were identified in 72.5% (37/51) of SUD cases. Notably, three VUSs, including one novel variant, demonstrated strong supporting evidence for pathogenicity, with each detected in a distinct case. Pathogenic or LP variants were detected in approximately 4% (2/51) of cases, indicating a probable genetic base for an arrhythmic or cardiac conduction disorder.

Despite the limited and non-representative sample size, this study provides initial insights into the genetic factors contributing to SUDs in young South Africans; however, the small cohort size, combined with a narrower gene panel, may have contributed to a potential underdiagnosis of SUD cases.

This study highlights the critical need for South Africa to expand molecular research into the causes of sudden unexplained deaths (SUDs). Future efforts should focus on large-scale studies utilizing comprehensive gene panels to deepen our understanding of the genetic factors contributing to SUDs in this population and to ensure that the findings deliver tangible benefits to the community.

### Key points

1. **Prevalence of Cardiac-Related Sudden Death:** Cardiac-related SUD cases (60–90% of all SUDs) are termed sudden cardiac deaths (SCD). These cases are a significant public health concern, with an estimated annual global incidence of 15–159 cases per 100,000 people. This study focused on post-mortem genetic testing, in order to identify genetic variants that could explain sudden unexpected deaths (SUD) in young individuals, with an emphasis on genetic variants linked to cardiac arrhythmogenic disorders.
2. **Study cohort and genetic findings:** The research analysed 51 unexplained SUD cases from South Africa and found 175 different missense variants. Although most variants were typed as likely benign, one was classified as pathogenic, and another as likely pathogenic. The latter two could both be linked to cardiac arrhythmogenic disorders.
3. **Underrepresentation of African cohorts:** The study highlights a significant gap in genetic research in African populations, particularly in the context of SUD. This underrepresentation emphasizes the need for more region-specific research.
4. **Case-specific findings:** In two of the study cases, genetic variants in the *SCN10A* and *KCNE1* genes were identified, with one variant linked to Brugada syndrome and another to long QT syndrome. These findings suggest that the genetic variations may have contributed to the sudden deaths in these individuals.

## Electronic supplementary material

Below is the link to the electronic supplementary material.

Supplementary Material 1

Supplementary Material 2

Supplementary Material 3

Supplementary Material 4

## References

[1] Heathfield LJ, Martin LJ, Ramesar R. Massively parallel sequencing in sudden unexpected death in infants: a case report in South Africa. Forensic Sci Int Genet. 2019;7(1):459–61. 10.1016/j.fsigss.2019.10.051.

[2] Shanks GW, Tester DJ, Ackerman JP, Simpson MA, Behr ER, White SM, Ackerman MJ. Importance of variant interpretation in whole-exome molecular autopsy: Population-based case series. Circulation. 2018;137(25):2705–15. 10.1161/circulationaha.117.031053. 29915097 10.1161/CIRCULATIONAHA.117.031053

[3] Sanchez O, Campuzano O, Fernandez-Falgueras A, Sarquella-Brugada G, Cesar S, Mademont I, Mates J, Perez-Serra A, Coll M, Pico F, Iglesias A, Tiron C, Allegue C, Carro E, Gallego MA, Ferrer-Costa C, Hospital A, Bardalet N, Borondo JC, Vingut A, Arbelo E, Brugada J, Castella J, Medallo J, Brugada R. Natural and undetermined sudden death: value of post-mortem genetic investigation. PLoS ONE. 2016;11(12):e0167358. 10.1371/journal.pone.0171893. 27930701 10.1371/journal.pone.0167358 PMC5145162

[4] Matthews E, Blair P, Sisodiya S, Jones S, Sebire N, Behr E, Fleming P. National registry for sudden unexpected deaths of infants and children in England: why do we need one and do families want one? Arch Dis Child. 2019;104(10):989–93. 10.1136/archdischild-2018-316542. 31005897 10.1136/archdischild-2018-316542 PMC6889686

[5] Campuzano O, Beltramo P, Fernandez A, Iglesias A, Garcia L, Allegue C, Sarquella-Brugada G, Coll M, Perez-Serra A, Mademont-Soler I, Mates J, Del Olmo B, Rodriguez A, Maciel N, Puigmule M, Pico F, Cesar S, Brugada J, Cuesta A, Gutierrez C, Brugada R. Molecular autopsy in a cohort of infants died suddenly at rest. Forensic Sci Int Genet. 2018;37:54–63. 10.1016/j.fsigen.2018.07.023. 30086531 10.1016/j.fsigen.2018.07.023

[6] Anderson JH, Tester DJ, Will ML, Ackerman MJ. Whole-exome molecular autopsy after exertion-related sudden unexplained death in the young. Circ Cardiovasc Genet. 2016;9(3):259–65. 10.1161/circgenetics.115.001370. 27114410 10.1161/CIRCGENETICS.115.001370

[7] Dempers JJ, du Toit-Prinsloo et al. A South African Perspective. Duncan J.R BRW, editor. Adelaide: The University of Adelaide Press; 2018. 30035954

[8] Bagnall RD, Weintraub RG, Ingles J, Duflou J, Yeates L, Lam L, Davis AM, Thompson T, Connell V, Wallace J, Naylor C, Crawford J, Love DR, Hallam L, White J, Lawrence C, Lynch M, Morgan N, James P, du Sart D, Puranik R, Langlois N, Vohra J, Winship I, Atherton J, McGaughran J, Skinner JR, Semsarian C. A prospective study of sudden cardiac death among children and young adults. N Engl J Med. 2016;374(25):2441–52. 10.1056/nejmoa1510687. 27332903 10.1056/NEJMoa1510687

[9] Lahrouchi N, Raju H, Lodder EM, Papatheodorou E, Ware JS, Papadakis M, Tadros R, Cole D, Skinner JR, Crawford J, Love DR, Pua CJ, Soh BY, Bhalshankar JD, Govind R, Tfelt-Hansen J, Winkel BG, van der Werf C, Wijeyeratne YD, Mellor G, Till J, Cohen MC, Tome-Esteban M, Sharma S, Wilde AAM, Cook SA, Bezzina CR, Sheppard MN, Behr ER. Utility of post-mortem genetic testing in cases of sudden arrhythmic death syndrome. J Am Coll Cardiol. 2017;69(17):2134–45. 10.1016/j.jacc.2017.02.046. 28449774 10.1016/j.jacc.2017.02.046 PMC5405216

[10] Christiansen SL, Hertz CL, Ferrero-Miliani L, Dahl M, Weeke PE, LuCamp, Ottesen GL, Frank-Hansen R, Bundgaard H, Morling N. Genetic investigation of 100 heart genes in sudden unexplained death victims in a forensic setting. Eur J Hum Genet. 2016;24(12):1797–802. 10.1038/ejhg.2016.118. 27650965 10.1038/ejhg.2016.118 PMC5117921

[11] Neubauer J, Lecca MR, Russo G, Bartsch C, Medeiros-Domingo A, Berger W, Haas C. Post-mortem whole-exome analysis in a large sudden infant death syndrome cohort with a focus on cardiovascular and metabolic genetic diseases. Eur J Hum Genet. 2017;25(4):404–9. 10.1038/ejhg.2016.199. 28074886 10.1038/ejhg.2016.199 PMC5386419

[12] Hertz CL, Christiansen SL, Ferrero-Miliani L, Fordyce SL, Dahl M, Holst AG, Ottesen GL, Frank-Hansen R, Bundgaard H, Morling N. Next-generation sequencing of 34 genes in sudden unexplained death victims in forensics and in patients with channelopathic cardiac diseases. Int J Legal Med. 2015;129(4):793–800. 10.1007/s00414-014-1105-y. 25467552 10.1007/s00414-014-1105-y

[13] Bailly C, Henriques S, Tsabedze N, Krause A. Role of family history and clinical screening in the identification of families with idiopathic dilated cardiomyopathy in Johannesburg, South Africa. S Afr Med J. 2019;109(9):673–8. 10.7196/samj.2019.v109i9.13936. 31635593 10.7196/SAMJ.2019.v109i9.13936

[14] Walsh R, Peters NS, Cook SA, Ware JS. Paralogue annotation identifies novel pathogenic variants in patients with Brugada syndrome and catecholaminergic polymorphic ventricular tachycardia. J Med Genet. 2014;51(1):35–44. 10.1136/jmedgenet-2013-101917. 24136861 10.1136/jmedgenet-2013-101917 PMC3888601

[15] Li Y, Wei J, Guo W, Sun B, Estillore JP, Wang R, Yoruk A, Roston TM, Sanatani S, Wilde AAM, Gollob MH, Roberts JD, Tseng ZH, Jensen HK, Chen SRW. Human RyR2 (ryanodine receptor 2) loss-of-function mutations: clinical phenotypes and in vitro characterization. Circ Arrhythm Electrophysiol. 2021;14(9):e010013. 10.1161/circep.121.010013. 34546788 10.1161/CIRCEP.121.010013

[16] Fowler ED, Zissimopoulos S. Molecular, subcellular, and arrhythmogenic mechanisms in genetic RyR2 disease. Biomolecules. 2022;12(8). 10.3390/biom12081030. 10.3390/biom12081030 PMC9394283 35892340

[17] Stutzman MJ, Kim CSJ, Tester DJ, Hamrick SK, Dotzler SM, Giudicessi JR, Miotto MC, Gc JB, Frank J, Marks AR, Ackerman MJ. Characterization of N-terminal RYR2 variants outside CPVT1 hotspot regions using patient iPSCs reveal pathogenesis and therapeutic potential. Stem Cell Rep. 2022;17(9):2023–36. 10.1016/j.stemcr.2022.07.002. 10.1016/j.stemcr.2022.07.002 PMC9481874 35931078

[18] Richards S, Aziz N, Bale S, Bick D, Das S, Gastier-Foster J. Standards and guidelines for the interpretation of sequence variants: a joint consensus recommendation of the American College of Medical Genetics and Genomics and the Association for Molecular Pathology. Genet Med. 2015;17:405–24. 10.1038/gim.2015.30. 25741868 10.1038/gim.2015.30 PMC4544753

[19] Xiao Z, Guo W, Sun B, Hunt DJ, Wei J, Liu Y, Wang Y, Wang R, Jones PP, Back TG, Chen SRW. Enhanced cytosolic Ca2 + activation underlies a common defect of central domain cardiac ryanodine receptor mutations linked to arrhythmias. J Biol Chem. 2016;291(47):24528–37. 10.1074/jbc.m116.756528. 27733687 10.1074/jbc.M116.756528 PMC5114406

[20] Woll KA, Van Petegem F. Calcium-release channels: structure and function of IP (3) receptors and ryanodine receptors. Physiol Rev. 2022;102(1):209–68. 10.1152/physrev.00033.2020. 34280054 10.1152/physrev.00033.2020

[21] Wu G, Ai T, Kim JJ, Mohapatra B, Xi Y, Li Z, Abbasi S, Purevjav E, Samani K, Ackerman MJ, Qi M, Moss AJ, Shimizu W, Towbin JA, Cheng J, Vatta M. Alpha-1-syntrophin mutation and the long-QT syndrome: a disease of sodium channel disruption. Circ Arrhythm Electrophysiol. 2008;1(3):193–201. 10.1161/CIRCEP.108.769224. 19684871 10.1161/CIRCEP.108.769224 PMC2726717

[22] Database GTHG. SNTA1 gene - Syntrophin Alpha 1 2023 [updated January 2023. Available from: https://www.genecards.org/cgi-bin/carddisp.pl?gene=SNTA1

[23] Lapidos KA, Kakkar R, McNally EM. The dystrophin glycoprotein complex: signaling strength and integrity for the sarcolemma. Circ Res. 2004;94(8):1023–31. 10.1161/01.res.0000126574.61061.25. 15117830 10.1161/01.RES.0000126574.61061.25

[24] Wang Q, Michalak M. Calsequestrin. Structure, function, and evolution. Cell Calcium. 2020;90:102242. 10.1016/j.ceca.2020.102242. 32574906 10.1016/j.ceca.2020.102242

[25] Lahat H, Pras E, Olender T, Avidan N, Ben-Asher E, Man O, Levy-Nissenbaum E, Khoury A, Lorber A, Goldman B, Lancet D, Eldar M. A missense mutation in a highly conserved region of CASQ2 is associated with autosomal recessive catecholamine-induced polymorphic ventricular tachycardia in Bedouin families from Israel. Am J Hum Genet. 2001;69(6):1378–84. 10.1086/324565. 11704930 10.1086/324565 PMC1235548

[26] Database GTHG. CASQ2 gene - Calsequestrin 2 2023 [updated January 2023. Available from: https://www.genecards.org/cgi-bin/carddisp.pl?gene=CASQ2&keywords=casq2

[27] Zhang L, Zhou F, Huang L, Wu Q, Zheng J, Wu Y, Yin K, Cheng J. Association of common and rare variants of SCN10A gene with sudden unexplained nocturnal death syndrome in Chinese Han population. Int J Legal Med. 2017;131(1):53–60. 10.1007/s00414-016-1397-1. 27272739 10.1007/s00414-016-1397-1

[28] Database GTHG. SCN10A gene - Sodium voltage-gated channel alpha subunit 10. 2023 [updated January 2023. Available from: https://www.genecards.org/cgi-bin/carddisp.pl?gene=SCN10A&keywords=scn10a

[29] Hu D, Barajas-Martinez H, Pfeiffer R, Dezi F, Pfeiffer J, Buch T, Betzenhauser MJ, Belardinelli L, Kahlig KM, Rajamani S, DeAntonio HJ, Myerburg RJ, Ito H, Deshmukh P, Marieb M, Nam GB, Bhatia A, Hasdemir C, Haissaguerre M, Veltmann C, Schimpf R, Borggrefe M, Viskin S, Antzelevitch C. Mutations in SCN10A are responsible for a large fraction of cases of Brugada syndrome. J Am Coll Cardiol. 2014;64(1):66–79. 10.1016/j.jacc.2014.04.032. 24998131 10.1016/j.jacc.2014.04.032 PMC4116276

[30] Abou Ziki MD, Seidelmann SB, Smith E, Atteya G, Jiang Y, Fernandes RG, Marieb MA, Akar JG, Mani A. Deleterious protein-altering mutations in the SCN10A voltage-gated sodium channel gene are associated with prolonged QT. Clin Genet. 2018;93(4):741–51. 10.1111/cge.13036. 28407228 10.1111/cge.13036 PMC5640462

[31] Behr ER, Savio-Galimberti E, Barc J, Holst AG, Petropoulou E, Prins BP, Jabbari J, Torchio M, Berthet M, Mizusawa Y, Yang T, Nannenberg EA, Dagradi F, Weeke P, Bastiaenan R, Ackerman MJ, Haunso S, Leenhardt A, Kaab S, Probst V, Redon R, Sharma S, Wilde A, Tfelt-Hansen J, Schwartz P, Roden DM, Bezzina CR, Olesen M, Darbar D, Guicheney P, Crotti L, Consortium UK, Jamshidi Y. Role of common and rare variants in SCN10A: results from the Brugada syndrome QRS locus gene discovery collaborative study. Cardiovasc Res. 2015;106(3):520–9. 10.1093/cvr/cvv042. 25691538 10.1093/cvr/cvv042 PMC4447806

[32] Clinvar. NM_000219.6(KCNE1):c.226G > A (p.Asp76Asn). National Institute of Health; 2024 (updated Aug 2024). Available from https://www.ncbi.nlm.nih.gov/clinvar/variation/13477/

[33] Zheng R, Thompson K, Obeng-Gyimah E, Alessi D, Chen J, Cheng H, McDonald TV. Analysis of the interactions between the C-terminal cytoplasmic domains of KCNQ1 and KCNE1 channel subunits. Biochem J. 2010;428(1):75–84. 10.1042/BJ20090977. 20196769 10.1042/BJ20090977 PMC2888147

[34] Database GTHG. KCNE1 gene - Potassium Voltage-Gated Channel Subfamily E Regulatory Subunit 1 2023 [updated January 2023. Available from: https://www.genecards.org/cgi-bin/carddisp.pl?gene=KCNE1&keywords=kcne

[35] Wu X, Larsson HP. Insights into Cardiac IKs (KCNQ1/KCNE1) channels regulation. Int J Mol Sci. 2020;21(24). 10.3390/ijms21249440. 10.3390/ijms21249440 PMC7763278 33322401

[36] Faridi R, Tona R, Brofferio A, Hoa M, Olszewski R, Schrauwen I, Assir MZK, Bandesha AA, Khan AA, Rehman AU, Brewer C, Ahmed W, Leal SM, Riazuddin S, Boyden SE, Friedman TB. Mutational and phenotypic spectra of KCNE1 deficiency in Jervell and Lange-Nielsen Syndrome and Romano-Ward Syndrome. Hum Mutat. 2019;40(2):162–76. 10.1002/humu.23689. 30461122 10.1002/humu.23689 PMC6328321

[37] Van Horn WD, Vanoye CG, Sanders CR. Working model for the structural basis for KCNE1 modulation of the KCNQ1 potassium channel. Curr Opin Struct Biol. 2011;21(2):283–91. 10.1016/j.sbi.2011.01.001. 21296569 10.1016/j.sbi.2011.01.001 PMC3070781

[38] Stiles MK, Wilde AAM, Abrams DJ, Ackerman MJ, Albert CM, Behr ER, Chugh SS, Cornel MC, Gardner K, Ingles J, James CA, Juang JJ, Kaab S, Kaufman ES, Krahn AD, Lubitz SA, MacLeod H, Morillo CA, Nademanee K, Probst V, Saarel EV, Sacilotto L, Semsarian C, Sheppard MN, Shimizu W, Skinner JR, Tfelt-Hansen J, Wang DW. 2020 APHRS/HRS expert consensus statement on the investigation of decedents with sudden unexplained death and patiets with sudden cardiac arrest, and of their families. J Arrhythm. 2021;37(3):481–534. 10.1016/j.hrthm.2020.10.010. 34141003 10.1002/joa3.12449 PMC8207384

[39] Laing N, Kraus SM, Shaboodien G, Ntusi NAB. An overview of the genetic basis of cardiovascular disease. South Afr Med J. 2019;109(6). 10.7196/samj.2019.v109i6.14069.

[40] Kauferstein S, Herz N, Scheiper S, Biel S, Jenewein T, Kunis M, Erkapic D, Beckmann BM, Neumann T. Relevance of molecular testing in patients with a family history of sudden death. Forensic Sci Int. 2017;276:18–23. 10.1016/j.forsciint.2017.04.001. 28472724 10.1016/j.forsciint.2017.04.001

