# Supplementary material for: Next generation sequencing: a possible answer to sudden unexplained deaths in a young South African cohort?
Source: Forensic Sci Med Pathol. 2025 Feb 3;21(3):1081–90. doi: 10.1007/s12024-025-00944-6 (PMC12491335; doi:10.1007/s12024-025-00944-6)
Supplement: Supplementary file 1 — Supplementary Material 1 [file 12024_2025_944_MOESM1_ESM.pdf]

Laboratory method

DNA quantification and dilution

- DNA was extracted from post mortem blood samples using the QIAamp DNA Blood Mini Kit from Qiagen (Hilden, Germany) and used according to prescribed guidelines provided by the manufacturer. Following DNA extraction, the initial concentration and purity ratio of all DNA samples were determined spectrophotometrically, by using the NanoDrop spectrophotometer (Thermo Scientific, Waltham, Massachusetts), and stored at -20°C until further use.

- Once all 66 case samples were collected, each DNA sample was fluorometrically quantified and diluted, (by using Low TE), to the required concentration of 4 ng/µl in a total diluted DNA volume of 5 µl (10 ng per primer pool), using the Qubit dsDNA HS Assay kit on the Qubit® 3.0 Fluorometer (ThermoFisher).

Amplification of DNA targets

The following reagents were used for PCR amplification of two primer pools:

20X AmpliSeq Sample ID Panel for Illumina
2X AmpliSeq DNA Panel Pool 1
2X AmpliSeq DNA Panel Pool 2
5X AmpliSeq HiFi Mix
DNA
Nuclease-free water

- To account for all 66 samples, a mastermix 1, containing 33 µl 20X AmpliSeq Sample ID Panel for Illumina and 330 µl of 2X AmpliSeq DNA Panel Pool 1 was prepared and mixed in a 1.5 ml tube. (Extra volume was prepared to account for small pipetting errors).

- An additional mastermix 2, containing 330 µl 5X AmpliSeq HiFi Mix and 165 µl nuclease-free water was prepared and mixed in a second 1.5 ml microcentrifuge tube.

- For each sample, a volume of 2.5 µl mastermix 2 was transferred to two wells of a new 96-well PCR plate. Next, 5.5 µl of mastermix 1 was added to the one well and 5 µl of 2X

AmpliSeq DNA Panel Pool 2 was added to the second well. This procedure was followed for all case samples. Lastly, 2.5 µl (4 ng/µl) of DNA was added to each sample's two prepared wells and pipetted to mix. The plate was sealed and briefly centrifuged.

➢ The plate was transferred to the Bio-Rad C1000 Touch PCR Thermal Cycler (Bio-Rad, Hercules, California) and ran on an AMP_DNA program according to the following settings:
  - Preheated lid, set to 105ºC with a reaction volume of 10 µl for two pools
  - Denaturation at 99ºC for two minutes
  - Sixteen cycles of:
    - 99ºC for 15 seconds
    - 60ºC for four minutes
  - Hold at 10ºC for 24 hours

<u>Partial digestion of amplicons</u>

Following PCR amplification, primer dimers and amplicons were partially digested by using FuPa reagent.

➢ The PCR plate (used in the previous step) was briefly centrifuged and unsealed.

➢ For each sample, the 10 µl of target amplification reactions in the two wells (prepared and amplified in the previous steps) were combined into the one well containing Pool 1.

➢ A total of 2 µl FuPa reagent was added to each 20 µl target amplification reaction for each sample.

➢ The plate was sealed, vortexed and briefly centrifuged before placing it in the thermal cycler, with the following conditions set:
  - Preheated lid, set to 105ºC and a set reaction volume of 22 µl
  - 50ºC for 10 minutes
  - 55ºC for 10 minutes
  - 62ºC for 20 minutes
  - Hold at 10ºC for one hour

Index ligation

The following reagents were used for ligating Index 1 (i7) and Index 2 (i5) adapters to each sample:

AmpliSeq CD Indexes for Illumina
DNA Ligase
Switch solution

- The library (PCR plate) was briefly centrifuged and unsealed, whereafter 4 µl of Switch Solution was added to each well containing digested amplicons.

- A volume of 2 µl AmpliSeq CD Indexes were added to each well, followed by the addition of 2 µl of DNA Ligase, also to each well.

- The library plate was sealed, vortexed and briefly centrifuged, whereafter it was placed on the thermocycler with the following preprogramed settings:
  - Preheated and set at 105ºC
  - 22ºC for 30 minutes
  - 68ºC for five minutes
  - 72ºC for five minutes
  - Hold at 10ºC for 24 hours

Library clean-up

Agencourt AMPure XP beads and freshly prepared 70% ethanol were used to clean up the library.

- Following brief centrifugation, the library plate was unsealed and 30 µl of AMPure XP beads was added to each well.

- The plate was briefly vortexed and centrifuged and incubated at room temperature for five minutes.

- The library plate was placed onto a magnetic stand until the mixture was clear, whereafter the plate was unsealed, and the entire supernatant removed and discarded from each well.

- The beads were washed by adding 150 µl of 70% ethanol to each well, incubated at room temperature until the solution was clear, followed by removing and discarding the supernatant from each well. This step was repeated once.

- After the washing step, the plate was sealed, vortexed and again placed on the magnetic stand and unsealed.

- All residual ethanol was removed from each well and air-dried on the magnetic stand for at least 10 minutes.

Second library amplification

For amplification, the following reagents were used to prepare an amplification mastermix:

**Reagent**	**Volume (µl)**
1X Lib Amp Mix	45
10X Library Amp Primers	5
**Total volume per reaction**	**50**

- The mastermix was briefly vortexed and centrifuged, whereafter the plate was removed from the magnetic stand and 50 µl of amplification mastermix was added to each library well.

- The plate was sealed, vortexed and briefly centrifuged, and then place onto the preprogramed C1000 Touch PCR Thermal Cycler (Bio-Rad) according to the following settings:
  - Preheated and set at 105ºC
  - 98ºC for two minutes
  - Seven cycles of:
    - 98ºC for 15 seconds
    - 64ºC for one minute
  - Hold at 10ºC for 24 hours

Second library clean-up

AMPure XP beads were also used for the second clean-up of the library:

- Following brief centrifugation, the library plate was unsealed and 25 µl of AMPure XP beads was added to each library-containing well.
- The library was once again quickly vortexed and centrifuged, followed by incubation at room temperature for five minutes.
- The plate was placed on a magnetic stand for at least five minutes, or until the liquid was clear.
- Next, the entire supernatant, containing the amplicon library, was transferred to a new plate.
- A volume of 60 µl of AMPure XP beads was added to each well containing the supernatant. The plate was sealed, vortexed briefly, followed by centrifugation.
- Following incubation at room temperature for five minutes, the plate was placed on the magnetic stand, again for five minutes, or until the liquid was clear.
- The plate was unsealed and the supernatant from each well was removed and discarded.
- The beads were washed by adding 150 µl of 70% ethanol to each well, incubated at room temperature until the solution is clear, followed by the removal of the supernatant from each well. This step was repeated once.
- All residual 70% ethanol was removed from each well and discarded, whereafter the plate was airdried on the magneticsstand for at least five minutes.
- The plate was removed from the magnetic stand, and 30 µl of Low TE was added to each well.
- The plate was vortexed and centrifuged, and then placed on a magnetic stand for at least five minutes.

- A total of 27 µl amplicon library-containing supernatant was transferred to a new LoBind PCR plate.

Assess library quality

To assess the quality of the library, the Agilent 2100 Bioanalyzer with the Agilent DNA 1000 Kit was used. This allowed for 12 samples to be processed, simultaneously, per DNA chip.

The following reagents were used for this procedure:

DNA ladder (yellow)
DNA markers (green)
DNA dye concentrate (blue)
DNA gel matrix (red)
Amplified library

- A volume of 25 µl DNA dye concentrate was added to a DNA gel matrix vial, vortexed and briefly centrifuged to spin it down.

- The solution was transferred to a spin filter, whereafter it was centrifuged at 2240 g for 15 minutes.

- A new DNA chip was placed in the chip priming station, and 9 µl of the prepared gel-dye mix was pipetted into the allocated, marked well.

- Next, the plunger, positioned at 1 ml, was pressed for 60 seconds before the clip was released and pulled back to its original position.

- Another 9 µl of gel-dye mixed was added to the other two allocated, marked wells.

- Next, 5 µl of DNA marker was added to all 12 sample well, as well as the ladder well.

- A total of 1 µl DNA ladder was added to its allocated, marked well, whereafter 1 µl of sample was added to its 12 allocated wells.

- The chip was horizontally placed in the adapter and vortexed for one minute at a speed of 650 x g, and finally run on the Bioanalyzer for measurements.

Library quantification

The following reagents were used for the quantification of the library:

Qubit® dsDNA HS reagent
Qubit® dsDNA HS buffer
Qubit® ds DNA HS Standard #1
Qubit® ds DNA HS Standard #2

- A Qubit working solution was prepared by diluting the Qubit® dsDNA HS Reagent 1:200 in Qubit® dsDNA HS Buffer. Enough working solution was prepared to reach the required 198 µl and190 µl for each sample and standard tube.

- Next, 190 µl of Qubit working solution was added to two tubes, followed by the addition of 10 µl of each DNA standard #1 and #2 to its corresponding tube.

- For library-containing samples, 198 µl of the Qubit working solution was added to each tube, followed by 2 µl of library-containing sample to each corresponding tube.

- With a total volume of 200 µl, each tube was quickly vortexed and incubated at room temperature for two minutes.

- Following incubation, sample concentration was measured using the Qubit® 3.0 Fluorometer. DNA standard #1 and #2 were used for calibration, whereafter the DNA concentration of all library-containing samples was measured.

Library dilution to starting concentration

The following formula was used to determine the molarity value of the pooled libraries:

$$\textbf{Molarity (nM)} = \frac{ng\,/\,\mu l \times 10^{6}}{660\ {}^{g}/_{mol} \times \text{average library size (bp)}}$$

- Using Low TE, each library pool was diluted (in a new LoBind PCR plate), to a starting concentration of 2 nM.

Library denaturation and dilution to final loading concentration

The following reagents were used to denature and dilute the libraries:

Fresh prepared 0.2 N NaOH dilution
HT1 buffer
Low TE buffer
200 mM Tris-HCl (pH 7.0)

- Equal volumes of each library were transferred to a 1.5 ml LoBind tube, whereafter it was vortexed and briefly centrifuged.

- A total of 10 µl of each library pool was added to a new tube, followed by 10 µl of 0.2 N NaOH. The samples were briefly vortexed and centrifuged, and incubated at room temperature for five minutes.

- Next, 10 µl of 200 mM Tris HCl was added to the tube containing pooled libraries, and vortexed, followed by brief centrifugation.

- A volume of 970 µl prechilled HT1 buffer was added, which resulted in a 20 pM denatured library.

- After vortex and brief centrifugation, prechilled HT1 buffer was used to further dilute the pooled libraries to a final loading concentration of 1.5 pM, up to a final volume of 1.3 ml. The tube was inverted to mix, briefly vortexed and then centrifuged.

Next generation sequencing

Pooled libraries were sequenced using the Illumina NextSeq Reagent kit (v2.5 300 cycles) on the Illumina NextSeq 550 platform. The Reagent kit contained the following:

Reagent cartridge
Buffer cartridge
Flow cell
HT1 buffer

- The reagent cartridge was thawed in a room temperature water bath for at least one hour, or until completely thawed.

- The flow cell package was unwrapped and set aside at room temperature for 30 minutes.

- Once removed from the plastic package, the surface of the flow cell was cleaned with a lint-free alcohol wipe, whereafter the glass was dried with lint-free lab tissue.

- A volume of 1.3 ml of prepared libraries (1.5 pM) was loaded into reservoir #10 on the Reagent cartridge.

- Finally, the Reagent cartridge, clean flow cell and buffer cartridge were loaded into the NextSeq system (each into their allocated compartments), whereafter cluster and sequencing was performed.

.
