# Supplementary material for: Next generation sequencing: a possible answer to sudden unexplained deaths in a young South African cohort?
Source: Forensic Sci Med Pathol. 2025 Feb 3;21(3):1081–90. doi: 10.1007/s12024-025-00944-6 (PMC12491335; doi:10.1007/s12024-025-00944-6)
Supplement: Supplementary file 2 — Supplementary Material 2 [file 12024_2025_944_MOESM2_ESM.pdf]

List of 49 genes included in the Ampliseq On-Demand DNA panel

Gene	Protein name	Chromosome	Accession number
ABCC9	ATP Binding Cassette Subfamily C Member 9	12	NM_020297.3
AKAP9	A-Kinase Anchoring Protein 9	7	NM_00575.4
ANK2	Ankyrin 2	4	NM_020977.3
ASPH	Aspartate Beta-Hydroxylase	8	NM_001164751.1
CACNA1C	Calcium Voltage-Gated Channel Subunit Alpha1 C	12	NM_199460.2
CACNA1D	Calcium Voltage-Gated Channel Subunit Alpha1 D	3	NM_000720.3
CACNA2D1	Calcium Voltage-Gated Channel Auxiliary Subunit Alpha2delta 1	7	NM_000722.2
CACNB2	Calcium Voltage-Gated Channel Auxiliary Subunit Beta 2	10	NM_201596.2
CALM1	Calmodulin 1	14	NM_006888
CALM2	Calmodulin 2	2	NM_001743
CALM3	Calmodulin 3	19	NM_005184.2
CASQ2	Calsequestrin 2	1	NM_001232.3
CAV3	Caveolin 3	3	NM_001234.4
CDH2	Cadherin 2	18	NM_001792.5
DPP6	Dipeptidyl Peptidase Like 6	7	NM_130797
EMILIN3	Elastin Microfibril Interfacer 3	20	NM_052846.2
GJA5	Gap Junction Protein Alpha 5	1	NM_181703.4
GPD1L	Glycerol-3-Phosphate Dehydrogenase 1 Like	3	NM_015141.3
HCN4	Hyperpolarization Activated Cyclic Nucleotide Gated Potassium Channel 4	15	NM_005477.2
KCNA5	Potassium Voltage-Gated Channel Subfamily A Member 5	12	NM_002234.4
KCND3	Potassium Voltage-Gated Channel Subfamily D Member 3	1	NM_004980.4
KCNE1	Potassium Voltage-Gated Channel Subfamily E Regulatory Subunit 1	21	NM_001127670.1
KCNE2	Potassium Voltage-Gated Channel Subfamily E Regulatory Subunit 2	21	NM_172201.2
KCNE3	Potassium Voltage-Gated Channel Subfamily E Regulatory Subunit 3	11	NM_005472.5
KCNE5	Potassium Voltage-Gated Channel Subfamily E Regulatory Subunit 5	X	NM_012282.4
KCNH2	Potassium Voltage-Gated Channel Subfamily H Member 2	7	NM_000238.3
KCNJ2	Potassium Inwardly Rectifying Channel Subfamily J Member 2	17	NM_000891.2
KCNJ5	Potassium Inwardly Rectifying Channel Subfamily J Member 5	11	NM_000890.3

Gene	Protein name	Chromosome	Accession number
KCNJ8	Potassium Inwardly Rectifying Channel Subfamily J Member 8	12	NM_004982.3
KCNQ1	Potassium Voltage-Gated Channel Subfamily Q Member 1	11	NM_000218.2
LMNA	Lamin A/C	1	NM_170707.2
NPPA	Natriuretic Peptide A	1	NM_012612.2
PKP2	Plakophilin 2	12	NM_004572.3
PLN	Phospholamban	6	NM_002667.3
PRKAG2	Protein Kinase AMP-Activated Non-Catalytic Subunit Gamma 2	7	NM_016203
RANGRF	RAN Guanine Nucleotide Release Factor	17	NM_016492.5
RYR2	Ryanodine Receptor 2	1	NM_001035.2
SAMD11	Sterile Alpha Motif Domain Containing 11	1	NM_152486.4
SCN10A	Sodium Voltage-Gated Channel Alpha Subunit 10	3	NM_006514.2
SCN1B	Sodium Voltage-Gated Channel Beta Subunit 1	19	NM_001321605
SCN2B	Sodium Voltage-Gated Channel Beta Subunit 2	11	NM_004588.5
SCN3B	Sodium Voltage-Gated Channel Beta Subunit 3	11	NM_018400.3
SCN4B	Sodium Voltage-Gated Channel Beta Subunit 4	11	NM_174934.3
SCN5A	Sodium Voltage-Gated Channel Alpha Subunit 5	3	NM_198056.2
SLMAP	Sarcolemma Associated Protein	3	NM_007159.2
SNTA1	Syntrophin Alpha 1	20	NM_003098.2
TNNT2	Troponin T2, Cardiac Type	1	NM_000364.4
TRDN	Triadin	6	NM_006073.3
TRPM4	Transient Receptor Potential Cation Channel Subfamily M Member 4	19	NM_017636.3
