# Supplementary material for: Next generation sequencing: a possible answer to sudden unexplained deaths in a young South African cohort?
Source: Forensic Sci Med Pathol. 2025 Feb 3;21(3):1081–90. doi: 10.1007/s12024-025-00944-6 (PMC12491335; doi:10.1007/s12024-025-00944-6)
Supplement: Supplementary file 3 — Supplementary Material 3 [file 12024_2025_944_MOESM3_ESM.pdf]

Case sample data sheet

Case number	Age	Sex	Cause of death	Activity at time of death
1	35y	Male	U/I	Exertion
2	19y	Male	U/I	Unknown
3	35y	Female	U/I	Unknown
4	20y	Female	U/I	Sleep
5	41y	Male	U/I	Unknown
6	35y	Male	U/I	Exertion
7	29y	Male	U/I	Sleep
8	31y	Male	U/I	Under severe stress
9	27y	Male	U/I	Exertion
10	24y	Male	U/I	Unknown
11	25y	Female	U/I	Rest
12	30y	Male	U/I	Rest
13	32y	Male	U/I	Sleep
14	16y	Male	U/I	Exertion
15	35y	Male	U/I	Sleep
16	18y	Female	U/I	Sleep
17	4y	Male	U/I	Unknown
18	38y	Male	U/I	Exertion
19	36y	Male	U/I	Rest
20	27y	Male	U/I	Unknown
21	26y	Male	U/I	Exertion
22	4y	Female	U/I	Sleep

Case number	Age	Sex	Cause of death	Activity at time of death
23	23y	Female	U/I	Sleep
24	23y	Male	U/I	Exertion
25	27y	Male	U/I	Rest
26	40y	Male	U/I (Possible HCM)	Rest
27	17y	Female	U/I	Rest
28	30y	Male	U/I	Unknown
29	33y	Male	U/I	Unknown
30	41y	Male	U/I Possible HCM / Arrhythmia	Sleep
31	21y	Female	U/I	Unknown
32	23y	Male	U/I	Rest
33	28y	Male	Unascertained at autopsy alone	Unknown
34	30y	Male	U/I	Rest
35	29y	Female	SCD (Possible DCM)	Rest
36	35y	Female	U/I	Rest
37	17y	Male	U/I	Exertion
38	21y	Female	U/I	Sleep
39	39y	Female	U/I	Sleep
40	32y	Male	U/I	Exertion
41	38y	Female	U/I	Exertion
42	35y	Male	U/I	Unknown
43	14y	Female	U/I	Sleep
44	35y	Male	U/I	Rest
45	32y	Female	U/I	Sleep
46	45y	Male	U/I	Unknown
47	13y	Female	U/I	Sleep

Case number	Age	Sex	Cause of death	Activity at time of death
48	44y	Male	U/I	Unknown
49	29y	Male	U/I	Exertion
50	29y	Male	U/I	Unknown
51	28y	Male	U/I	Unknown
Abbreviations: U/I = under investigation; w = weeks; y = years				
