# Supplementary material for: Next generation sequencing: a possible answer to sudden unexplained deaths in a young South African cohort?
Source: Forensic Sci Med Pathol. 2025 Feb 3;21(3):1081–90. doi: 10.1007/s12024-025-00944-6 (PMC12491335; doi:10.1007/s12024-025-00944-6)
Supplement: Supplementary file 4 — Supplementary Material 4 [file 12024_2025_944_MOESM4_ESM.pdf]

List of 175 missense variants identified among the total study cohort

Gene	Chromosome	Variation g.	Variation c.	Variation p.	rs number	Predicted effect
SAMD11	chr1	865694	c.232C>T	p.H78Y	rs9988179	LB
KCNQ1	chr11	2610079	c.1388G>C	p.S463T	rs184636161	LB
CACNA1C	chr12	2742849	c.3883A>G	p.I1295V	rs114851656	LB
CACNA1C	chr12	2788878	c.5360C>T	p.T1787M	rs192749597	LB
CACNA1C	chr12	2788901	c.5383G>A	p.G1795R	rs111298509	B
CACNA1C	chr12	2791130	c.5459C>T	p.P1820L	rs10848683	B
CACNA1C	chr12	2791132	c.5461A>G	p.M1821V	rs10774053	B
CACNA1C	chr12	2791205	c.5534A>G	p.K1854R	rs10774054	B
CACNA1C	chr12	2794937	c.5666C>T	p.T1889M	rs201777030	B
KCNQ1	chr11	2869129	c.1546G>A	p.G516S	rs1800172	LB
KCNQ1	chr11	2869144	c.1561G>A	p.V521I	rs34150427	B
KCNA5	chr12	5153947	c.634C>T	p.R212C	rs77281462	VUS
KCNA5	chr12	5154173	c.860C>A	p.A287E	rs144246051	LB
KCNA5	chr12	5154431	c.1118G>C	p.G373A	rs1219418345	VUS
KCNA5	chr12	5154463	c.1150G>A	p.G384R	rs76709779	LB
KCNA5	chr12	5154925	c.1612G>C	p.E538Q	rs528221767	LB
KCNA5	chr12	5155046	c.1733G>A	p.R578K	rs12720445	B
KCNE3	chr11	6525592	c.68C>T	p.R23Q	rs35771371	LB
RANGRF	chr17	8192742	c.361C>G	p.R121G	rs773051695	LB
CAV3	chr3	8787263	c.166G>A	p.G56S	rs72546667	VUS
CAV3	chr3	8787330	c.233C>T	p.T78M	rs72546668	VUS
NPPA	chr1	11907648	c.94C>T	p.V32M	rs5063	B
CACNB2	chr10	18803444	c.510G>T	p.K170N	rs199539261	VUS
CACNB2	chr10	18803951	c.713G>A	p.G238D	rs142899184	LB
CACNB2	chr10	18827163	c.1357C>T	p.L453F	rs145638628	VUS
CACNB2	chr10	18828223	c.1553A>C	p.E518A	rs138060429	LB

Gene	Chromosome	Variation g.	Variation c.	Variation p.	rs number	Predicted effect
CACNB2	chr10	18828606	c.1936C>T	p.R646W	rs546669133	VUS
CACNB2	chr10	18828635	c.1803T>G	p.D601E	rs58225473	B
KCNJ8	chr12	21918931	c.1347A>G	p.V334A	rs34811413	B
ABCC9	chr12	21995312	c.3409C>T	p.V1137I	rs147895473	B
ABCC9	chr12	22017410	c.2200C>T	p.V734I	rs61688134	VUS
ABCC9	chr12	22040762	c.1909C>T	p.V637I	rs113542001	VUS
CACNA2D1	chr7	24890157	c.16C>T	p.L6F	rs1974332	LB
CDH2	chr18	25532304	c.2441T>C	p.N814S	rs2289664	LB
CDH2	chr18	25593655	c.298A>G	p.S100P	rs183606230	VUS
CDH2	chr18	25593694	c.352C>T	p.A118T	rs17445840	B
CDH2	chr18	25727748	c.61C>T	p.A21T	rs25727748	LB
SNTA1	chr20	32005726	c.500T>C	p.K167R	rs932909554	VUS
SNTA1	chr20	32026826	c.317C>T	p.R106Q	rs75025585	LB
GPD1L	chr3	32207381	c.1035G>T	p.Q345H	rs780760018	VUS
PKP2	chr12	32949140	c.2392T>C	p.T798A	rs112592855	LB
PKP2	chr12	32977026	c.1759C>T	p.V587I	rs146102241	VUS
PKP2	chr12	32996206	c.1420C>T	p.A474T	rs138538072	LB
PKP2	chr12	33003888	c.1190A>T	p.I397N	rs772334698	VUS
PKP2	chr12	33021934	c.1097A>G	p.L336P	rs1046116	B
PKP2	chr12	33049457	c.324C>A	p.S70I	rs75909145	LB
SCN1B	chr19	35524607	c.412G>A	p.V138I	rs72558029	LB
SCN1B	chr19	35524824	c.629T>C	p.L210P	rs55742440	B
SCN1B	chr19	35524939	c.744C>A	p.S248R	rs67701503	B
SCN1B	chr19	35524944	c.749G>C	p.R250T	rs67486287	LB
KCNE1	chr21	35821707	c.226C>T	p.D76N	rs74315445	P
KCNE1	chr21	35821821	c.112T>C	p.S38G	rs1805127	B
SCN5A	chr3	38591856	c.6007C>T	p.D2003N	rs376697724	VUS
SCN5A	chr3	38603947	c.3922G>A	p.L1308F	rs41313031	VUS

Gene	Chromosome	Variation g.	Variation c.	Variation p.	rs number	Predicted effect
SCN5A	chr3	38620907	c.3308G>T	p.S1103Y	rs7626962	LB
SCN5A	chr3	38620953	c.3262C>T	p.A1088T	rs369704754	VUS
SCN5A	chr3	38645378	c.1715G>A	p.A572L	rs36210423	LB
SCN5A	chr3	38645379	c.1714C>A	p.A572L	rs36210423	LB
SCN5A	chr3	38645420	c.1673T>C	p.H558R	rs1805124	LB
SCN5A	chr3	38645522	c.1571G>T	p.S524Y	rs41313691	LB
SCN5A	chr3	38651303	c.856C>A	p.A286S	rs61746118	LB
SCN5A	chr3	38651342	c.817T>C	p.M273V	Novel	LB
SCN5A	chr3	38674699	c.152G>A	p.R34C	rs6791924	LB
SCN10A	chr3	38739054	c.5657G>A	p.A1886	rs142653846	LB
SCN10A	chr3	38739574	c.5137T>C	p.M1713V	rs6599241	B
SCN10A	chr3	38739622	c.5089C>T	p.V1697I	rs77804526	B
SCN10A	chr3	38743380	c.4607G>C	p.T1536R	rs778754301	VUS
SCN10A	chr3	38760281	c3544C>A	p.V1182L	rs1396134485	LB
SCN10A	chr3	38763835	c.3427C>T	p.V1141M	rs112412281	VUS
SCN10A	chr3	38764998	c.3275A>G	p.L1092P	rs12632942	B
SCN10A	chr3	38766675	c.3218A>G	p.V1073A	rs6795970	B
SCN10A	chr3	38768300	c.2884T>C	p.I962V	rs57326399	B
SCN10A	chr3	38770193	c.2480C>G	p.W827S	rs1273210195	VUS
SCN10A	chr3	38793940	c.1525A>G	p.S509P	rs7630989	B
SCN10A	chr3	38793943	c.1522G>A	p.R508W	rs112774699	VUS
SCN10A	chr3	38812827	c.542T>G	p.E181A	rs142203439	VUS
SCN10A	chr3	38835462	c.40G>A	p.R14C	rs750771811	LP
EMILIN3	chr20	39990711	c.1498G>A	p.R500W	rs61739310	LB
TRPM4	chr19	49671207	c.301G>A	p.A101T	rs113984787	B
TRPM4	chr19	49671228	c.322C>T	p.R108C	rs115335683	LB
TRPM4	chr19	49685947	c.1376G>A	p.R459H	rs142312281	LB
**Gene**	**Chromosome**	**Variation g.**	**Variation c.**	**Variation p.**	**rs number**	**Predicted effect**

TRPM4	chr19	49693976	c.2156G>A	p.R719Q	rs78381230	LB
CACNA1D	chr3	53834369	c.5077G>A	p.E1693K	rs147973409	LB
CACNA1D	chr3	53835422	c.5438G>A	p.R1813Q	rs143003364	LB
CACNA1D	chr3	53839116	c.5752G>A	p.V1918Q	rs142184099	LB
SLMAP	chr3	57911641	c.943G>A	p.G315S	rs757046462	VUS
ASPH	chr8	62475338	c.1402C>T	p.G468R	rs61731238	LB
ASPH	chr8	62489332	c.1061T>C	p.Q354R	Novel	LB
ASPH	chr8	62496504	c.974C>A	p.R325M	rs6995412	VUS
ASPH	chr8	62546280	c.809C>T	p.S270N	rs111708484	LB
ASPH	chr8	62555970	c.645T>A	p.E215D	rs138586020	LB
ASPH	chr8	62577854	c.634A>T	p.Y212N	rs377016597	LB
ASPH	chr8	62577914	c.574A>G	p.S192P	rs762260016	LB
HCN4	chr15	73615097	c3337T>C	p.M1113V	rs142735148	B
HCN4	chr15	73615603	c.2831G>A	p.A994V	rs144450232	B
AKAP9	chr7	91603115	c.139C>T	p.H47Y	rs35669569	LB
AKAP9	chr7	91630532	c.1337G>A	p.R446Q	rs60031334	LB
AKAP9	chr7	91630603	c.1372G>C	p.A458P	rs143894795	B
AKAP9	chr7	91630620	c.1389G>T	p.M463I	rs6964587	B
AKAP9	chr7	91659259	c.4199T>C	p.M1400T	rs73407505	LB
AKAP9	chr7	91670136	c.4841G>A	p.R1614Q	rs2230768	B
AKAP9	chr7	91670172	c.4913T>A	p.L1638Q	Novel	LB
AKAP9	chr7	91708472	c.7025A>G	p.K2342R	Novel	LB
AKAP9	chr7	91708898	c.7451A>G	p.K2484R	rs35759833	LB
AKAP9	chr7	91712698	c.8375A>G	p.N2792S	rs6960867	B
AKAP9	chr7	91712808	c.8485G>A	p.E2829K	rs149946443	B
AKAP9	chr7	91714911	c.8935C>T	p.P2979S	rs1063242	LB
AKAP9	chr7	91726202	c.9929G>A	p.R3310Q	rs78351282	LB
AKAP9	chr7	91726522	c.10225C>T	p.R3409C	rs146495719	LB
**Gene**	**Chromosome**	**Variation g.**	**Variation c.**	**Variation p.**	**rs number**	**Predicted effect**

AKAP9	chr7	91726604	c.10331A>G	p.Q3444R	rs34956633	LB
AKAP9	chr7	91729127	c.10840A>G	p.M3614V	rs34327395	B
ANK2	chr4	114274492	c.4763	p.R1588K	Novel	LB
ANK2	chr4	114276255	c.6481A>C	p.K2161E	Novel	LB
ANK2	chr4	114276408	c.6679GA	p.G2227S	rs61734478	B
ANK2	chr4	114276781	c.7052C>T	p.A2351V	rs61734477	B
ANK2	chr4	114276880	c.7151T>C	p.V2369A	rs28377576	LB
ANK2	chr4	114277605	c.7831	p.Y2611H	rs35338364	B
ANK2	chr4	114277689	c.7915C>G	p.H2639D	rs529384341	LB
ANK2	chr4	114277870	c.8096T>C	p.M2699T	Novel	LB
ANK2	chr4	114278277	c.8503C>T	p.P2835S	rs3733617	B
ANK2	chr4	114278835	c.9061G>A	p.A3021T	rs74348333	LB
ANK2	chr4	114279228	c.9454A>G	p.T3152A	rs61741040	LB
ANK2	chr4	114288907	c.11218C>A	p.L3740I	rs35530544	LB
ANK2	chr4	114290816	c.11465G>C	p.G3822A	rs79577190	LB
ANK2	chr4	114294251	c.11616C>G	p.D3872E	rs768755447	LB
CASQ2	chr1	116243925	924A>T	p.D308E	rs776130201	VUS
CASQ2	chr1	116245602	c.954C>G	p.W318C	Novel	VUS
CASQ2	chr1	116247854	c.898C>T	p.D300N	rs376147306	VUS
CASQ2	chr1	116269619	c.731T>C	p.H244R	rs28730716	LB
CASQ2	chr1	116310967	c.196T>C	p.T66A	rs4074536	B
TRDN	chr6	123539749	c.2187C>A	p.Q729H	rs373439044	VUS
TRDN	chr6	123580777	c.1862T>G	p.E621A	rs1211286909	LB
TRDN	chr6	123594486	c.1620T>C	p.I540M	rs7771303	B
TRDN	chr6	123637602	c.1510C>T	p.G504S	rs150531306	LB
TRDN	chr6	123658776	c.1408G>T	p.L470M	rs6569336	B
TRDN	chr6	123687288	c.1313A>C	p.I438S	rs2873479	B
TRDN	chr6	123696766	c.1257G>T	p.D419E	rs17737379	B
**Gene**	**Chromosome**	**Variation g.**	**Variation c.**	**Variation p.**	**rs number**	**Predicted effect**

TRDN	chr6	123699019	c.1211A>C	p.V404G	rs28494009	B
TRDN	chr6	123714778	c.1096C>T	p.A366T	rs35047281	B
TRDN	chr6	123759243	c.1016C>T	p.S339N	rs35766971	B
TRDN	chr6	123786108	c.814G>A	p.P272S	rs549030753	LB
TRDN	chr6	123824902	c.755T>C	p.D252G	rs969285752	LB
TRDN	chr6	123824915	c.742T>C	p.K248E	Novel	LB
TRDN	chr6	123824918	c739G>C	p.P247A	rs1340220194	LB
TRDN	chr6	123833457	c.601G>C	p.L201V	rs6902416	B
TRDN	chr6	123868506	c.403C>T	p.E135K	rs192289289	B
TRDN	chr6	123869607	c.383G>C	p.T128S	rs9490809	B
TRDN	chr6	123869716	c.274C>T	p.V92I	rs34808221	B
KCNJ5	chr11	128781893	c.725G>A	p.R242Q	rs746240972	VUS
KCNJ5	chr11	128782012	c.844C>G	p.Q282E	rs7102584	B
KCNH2	chr7	150644005	c.3290A>G	p.V1097A	rs1484012284	LB
KCNH2	chr7	150644066	c.2209C>T	p.A737T	rs201382073	VUS
KCNH2	chr7	150644883	c.2488G>A	p.P830S	rs899224669	LB
KCNH2	chr7	150652572	c.20T>C	p.K7R	rs145819084	LB
KCNH2	chr7	150655521	c.542C>T	p.R181Q	rs41308954	LB
KCNH2	chr7	150671921	c.185C>T	p.R62Q	rs199473664	LB
PRKAG2	chr7	151478454	c.250G>A	p.R84W	rs61746358	VUS
PRKAG2	chr7	151573647	c.59C>A	p.S20I	rs116605521	LB
DPP6	chr7	154379517	c.785C>T	p.S262L	rs35392762	B
DPP6	chr7	154598774	c.1426A>G	p.S476G	Novel	LB
DPP6	chr7	154645534	c.1519A>C	p.K507Q	rs140460765	VUS
DPP6	chr7	154667632	c.1900A>G	p.S634G	Novel	LB
DPP6	chr7	154667692	c.1768G>A	p.G590S	rs150218787	LB
DPP6	chr7	154684153	c.2369T>C	p.L790P	rs3734960	LB
LMNA	chr1	156106187	c.1340A>G	p.E447G	Novel	LB
**Gene**	**Chromosome**	**Variation g.**	**Variation c.**	**Variation p.**	**rs number**	**Predicted effect**

TNNT2	chr1	201330429	c.749T>C	p.K250R	rs3730238	LB
TNNT2	chr1	201331240	c.604G>A	p.A206T	rs150008205	LB
RYR2	chr1	237619942	c.1519G>A	p.V507I	rs16835270	LB
RYR2	chr1	237711759	c.2935G>T	p.A979S	rs202015519	VUS
RYR2	chr1	237755076	c.4198A>G	p.S1400G	rs56229512	LB
RYR2	chr1	237778084	c.5656G>A	p.G1886S	rs3766871	LB
RYR2	chr1	237780752	c.5882A>G	p.K1961R	rs772508255	LB
RYR2	chr1	237813249	c.7585A>G	p.T2529A	Novel	LB
RYR2	chr1	237819159	c.8004G>C	p.C2668W	RCV004521091.1	VUS
RYR2	chr1	237841390	c.8873A>G	p.Q2985R	rs34967813	LB
